# Supplementary material for: Estimating the Distribution of Japanese Encephalitis Vectors in Australia Using Ecological Niche Modelling
Source: Trop Med Infect Dis. 2022 Nov 22;7(12):393. doi: 10.3390/tropicalmed7120393 (PMC9782987; doi:10.3390/tropicalmed7120393)
Supplement: Supplementary file 1 [file tropicalmed-07-00393-s001.zip › tropicalmed-1944001-Supplementary.pdf]

Supplementary Material

Here we present Supplementary Materials- Table S1 followed by Figure S1. Table S1 is a compilation of sample average values calculated by Maxent based on occurrence inputs and relevant environmental variables. Table S1 also serves as a definitions glossary for environmental variables listed in Figure 2a-2c. Note: only environmental variables with less than 90% correlation were included in Maxent simulations.

Table S1. Climate variables included in Maxent analysis, abbreviated file name and average values (units vary) for each vector species based on location data and relevant climate layer. Variable descriptions were obtained from the supporting file, 9sClimateMethodsSummary.pdf on the CSIRO Data Access Portal for the 9s climatology for continental Australia 1976-2005: Summary variables with elevation and radiative adjustment. .

Environmental Variable	Abbreviation	Average Values		
		Cx. annulirostris	*Cx. quinquefasciatus*	*Cx. sitiens*
Aridity Index				
Minimum monthly aridity index (proportion)	ADI	0.199	0.236	0.281
Mean annual aridity index (annual precipitation/annual potential evaporation) (proportion)	ADM	0.767	0.730	0.968
Maximum monthly aridity index (proportion)	ADX	2.150	2.661	2.476
Evaporation				
Annual total actual evapotranspiration (terrain scaled using MODIS) (mm)	EAA	806.602	725.109	966.189
Annual potential evaporation (mm)	EPA	1426.067	1283.282	1341.709
Minimum monthly potential evaporation	EPI	55.645	39.335	54.933
Maximum monthly potential evaporation (mm)	EPX	180.320	178.638	163.685
Precipitation				
Maximum monthly precipitation (mm)	PTX	206.702	154.127	241.677
Minimum Temperature				
Minimum temperature – monthly minimum (C)	TNI	10.323	7.975	11.563
Temperature Range				
Annual temperature range (C) (TXX – TNI)	TRA	22.172	22.724	18.858
Minimum monthly mean diurnal temperature (C)	TRI	9.312	9.286	7.976
Maximum monthly mean diurnal temperature (C)	TRX	13.740	13.449	11.717
Maximum Temperature				
Maximum temperature – monthly maximum (C)	TXX	32.495	30.700	30.420
Water Deficit				
Annual atmospheric water deficit (annual precipitation – annual potential evaporation) (mm)	WDA	-351.781	-365.283	-48.845
Maximum monthly atmospheric water deficit (precipitation – potential evaporation) (mm)	WDX	99.848	78.395	128.573

Additional niche models are included for ease of interpretation. These niche models were created using the manual interval data classification method in ArcMap version 10.7 to separate geographical areas by very low, low, medium and high probability of presence for each species. Manual interval inputs were based off initial Jenks natural breaks classifications but were adjusted to be the same for all species' models [1].

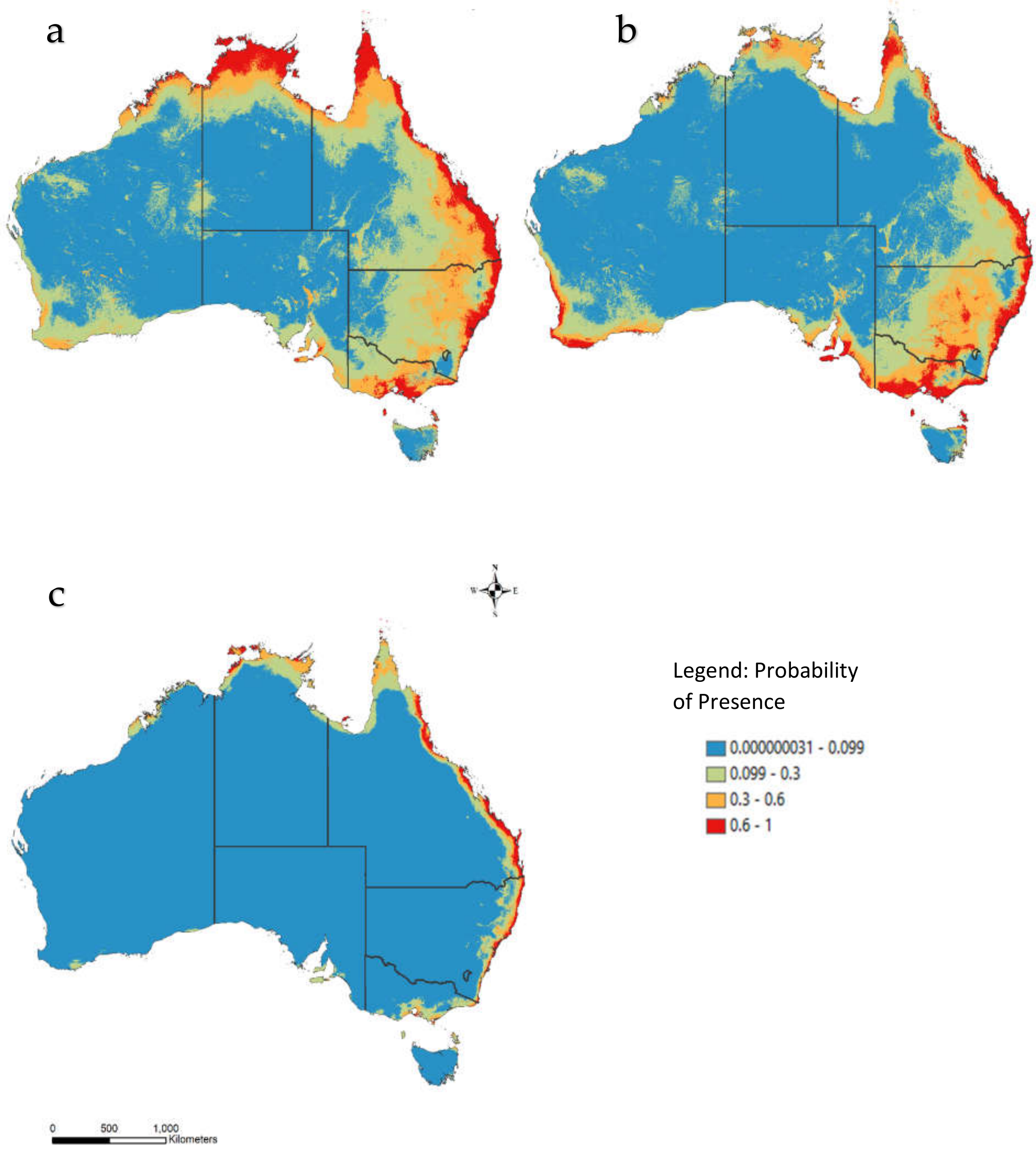

Figure S1. Ecological niche models for *Culex (Cx.) annulirostris* (a), *Cx. quinquefasciatus* (b), and *Cx. sitens* (c) in Australia classified from very low (blue), low (green), medium (yellow) to high (red) probability of species presence [see legend]. Australian states are labelled in the main manuscript.

We have also added ENMs created using k-fold cross-validation techniques in Maxent where k = 10 for further analysis. When using multiple replicates, Maxent output includes an uncertainty map based on the standard deviation between all model simulations.

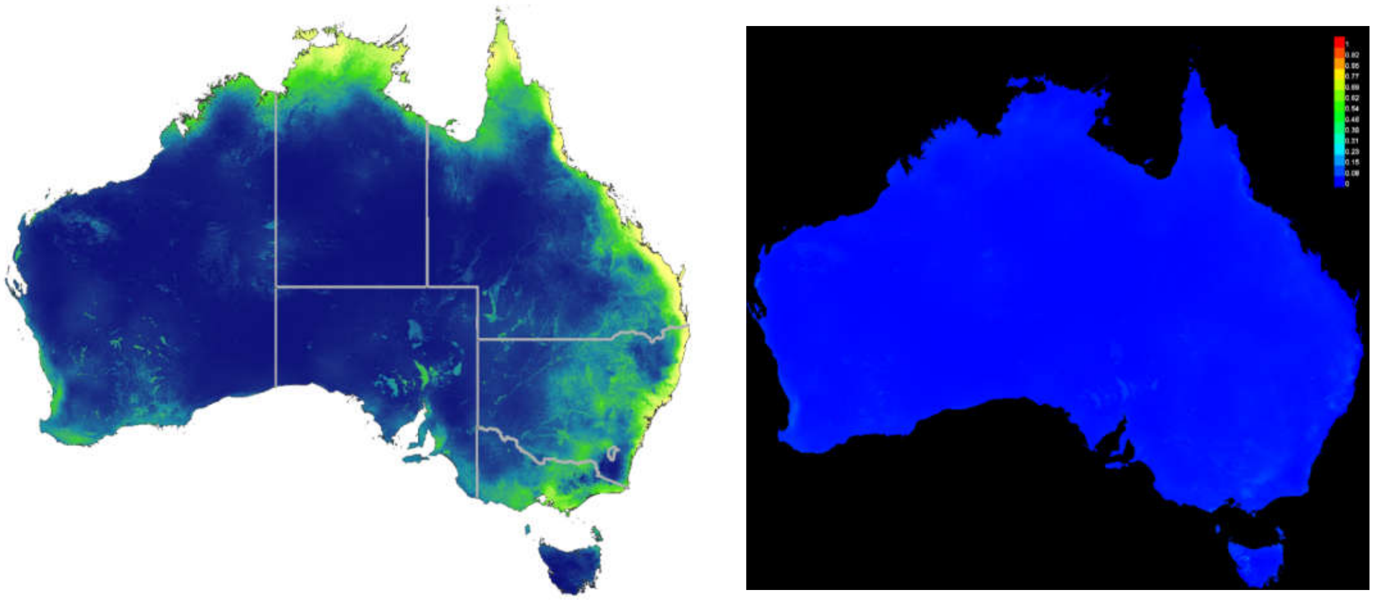

Legend: Estimated probability of presence for each vector species

a

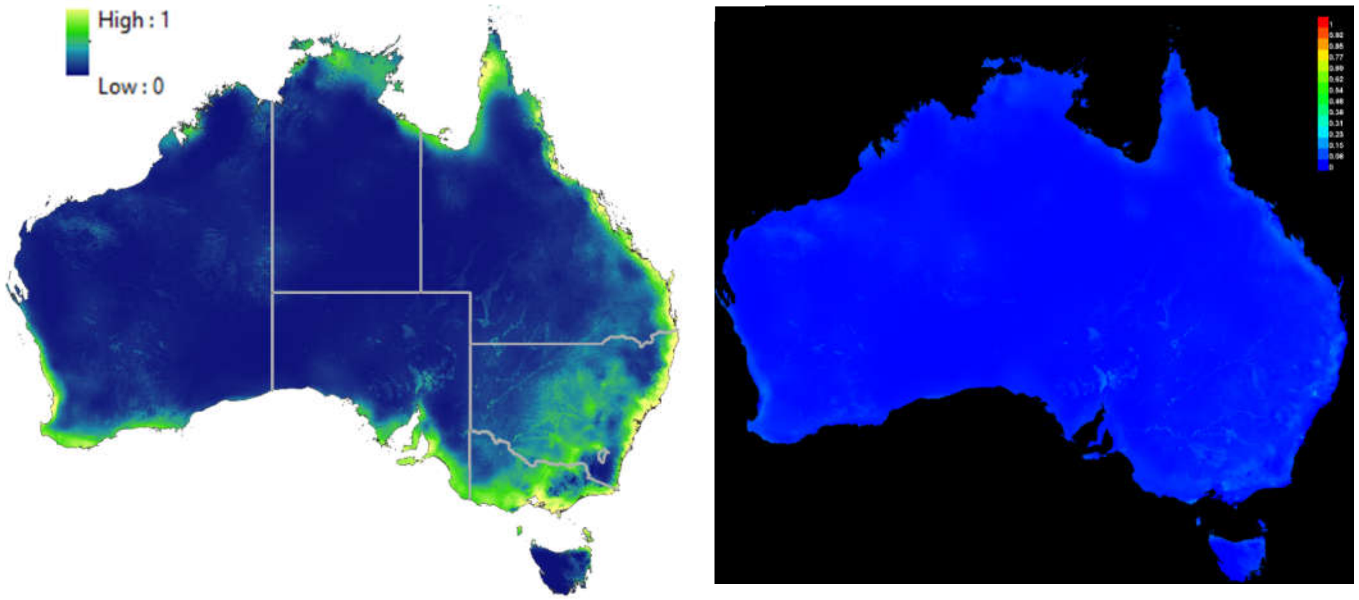

b

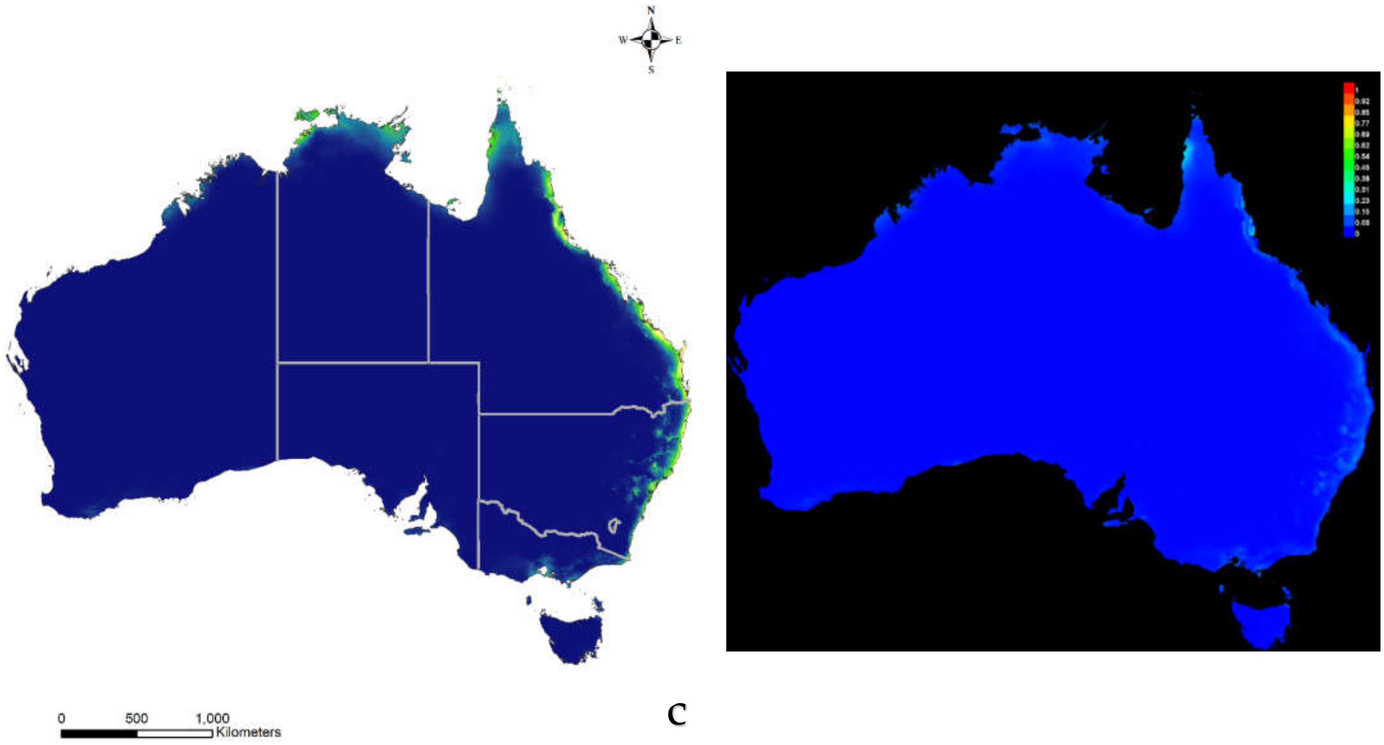

C

Figure S2. Cross-validation (left) and uncertainty (right) map outputs using the cross-validation options in Maxent where k was set to ten replicates. Cross-validation outputs represent the estimated probability of presence for *Cx. annulirostris* (a), *Cx. quinquefasciatus* (b) and *Cx. sitiens* (c) from 0 to 1, where 1 is equal to a 100% probability of presence. Uncertainty maps display standard deviation values between model replicates and range from 0 to 1, where 1 is considered an area of high uncertainty due to high levels of standard deviation. Areas of high uncertainty should be considered unreliable estimate predictions.

To investigate differences between the continental niche models estimated in this review and global occupied niches, occurrence records for each of the three mosquito species included in our study were downloaded from the Global Biodiversity Information Facility and environmental climate data from WorldClim to assess the estimated niche at a global level. The density of occurrence points noted for various temperature levels were then plotted. *Culex (Cx.) annulirostris* and *Cx. sitiens* populations in Australia appear to align within the suitable temperature ranges found amongst other population of these species' distributions worldwide. However, it appears as though our data predicts *Cx. quinquefasciatus* to exist among lower suitable temperature ranges than trends noted among global populations.

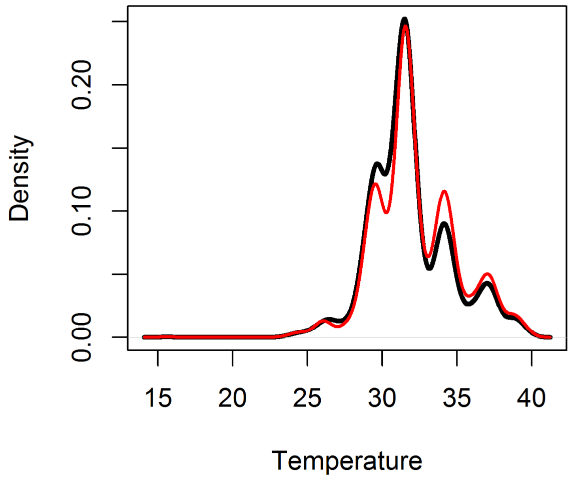

(a)

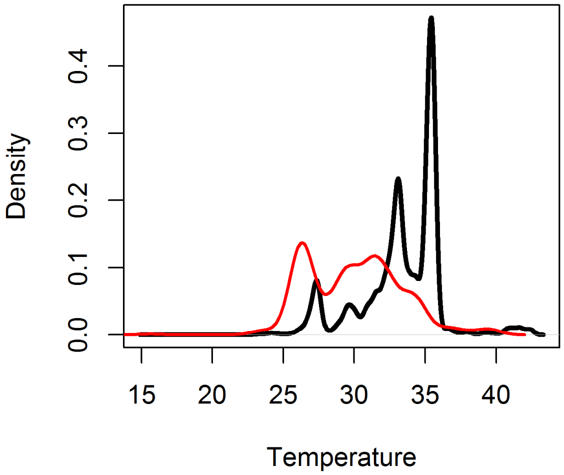

(b)

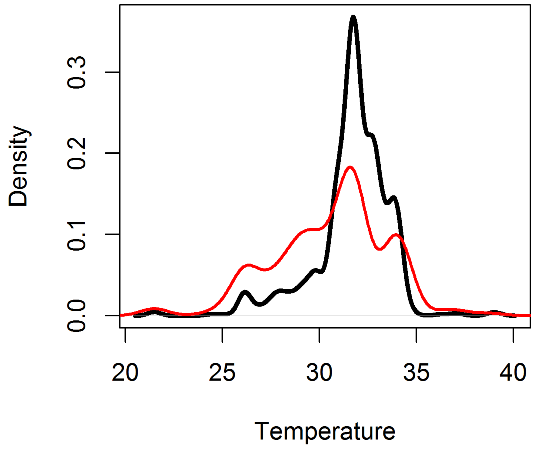

(c)

Figure S3. Comparisons of the observed temperature distributions from the Australian mosquito data used in the paper and global datasets for Culex (Cx.) annulirostris (a), Cx. quinquefasciatus (b) and Cx. sitiens (c).

References

1 esri. "Data classification methods." esri. https://pro.arcgis.com/en/pro-app/latest/help/mapping/layer-properties/data-classification-methods.htm (accessed 10 September 2022).
